# Peripheral loss of EphA4 ameliorates TBI-induced neuroinflammation and tissue damage

**DOI:** 10.1186/s12974-019-1605-2

**Published:** 2019-11-11

**Authors:** Elizabeth A. Kowalski, Jiang Chen, Amanda Hazy, Lauren E. Fritsch, Erwin Kristobal Gudenschwager-Basso, Michael Chen, Xia Wang, Yun Qian, Mingjun Zhou, Matthew Byerly, Alicia M. Pickrell, John B. Matson, Irving Coy Allen, Michelle H. Theus

**Affiliations:** 1 Department of Biomedical Sciences and Pathobiology, College of Veterinary Medicine, Virginia Tech, Blacksburg, VA 24061 USA; 2 Graduate Program in Translational Biology, Medicine, and Health, Virginia Tech, Blacksburg, VA 24061 USA; 3 Department of Mechanical Engineering, Virginia Tech, Blacksburg, VA 24061 USA; 4 Center for Drug Discovery, Virginia Tech, Blacksburg, VA 24061 USA; 5 School of Neuroscience, Virginia Tech, Blacksburg, VA 24061 USA; 6 Department of Chemistry, Virginia Tech, Blacksburg, VA 24061 USA; 7 Center for Regenerative Medicine, Virginia-Maryland College of Veterinary Medicine, Blacksburg, VA 24061 USA

**Keywords:** Inflammation, Traumatic brain injury, Interleukin-6, Akt, Endothelial cells, Monocyte/macrophage polarization

## Abstract

**Background:**

The continuum of pro- and anti-inflammatory response elicited by traumatic brain injury (TBI) is suggested to play a key role in the outcome of TBI; however, the underlying mechanisms remain ill -defined.

**Methods:**

Here, we demonstrate that using bone marrow chimeric mice and systemic inhibition of EphA4 receptor shifts the pro-inflammatory milieu to pro-resolving following acute TBI.

**Results:**

EphA4 expression is increased in the injured cortex as early as 2 h post-TBI and on CX3CR1^gfp^-positive cells in the peri-lesion. Systemic inhibition or genetic deletion of EphA4 significantly reduced cortical lesion volume and shifted the inflammatory profile of peripheral-derived immune cells to pro-resolving in the damaged cortex. These findings were consistent with in vitro studies showing EphA4 inhibition or deletion altered the inflammatory state of LPS-stimulated monocyte/macrophages towards anti-inflammatory. Phosphoarray analysis revealed that EphA4 may regulate pro-inflammatory gene expression by suppressing the mTOR, Akt, and NF-κB pathways. Our human metadata analysis further demonstrates increased *EPHA4* and pro-inflammatory gene expression, which correlates with reduced AKT concurrent with increased brain injury severity in patients.

**Conclusions:**

Overall, these findings implicate EphA4 as a novel mediator of cortical tissue damage and neuroinflammation following TBI.

## Background

Traumatic brain injury (TBI) represents a leading cause of long-term neurological disability. Physical trauma to the brain initiates a complex cascade of events that include vascular damage, ischemia, excitotoxicity, inflammation, and neuronal loss [1–3]. Pharmacological targeting of secondary injury processes such as neuroinflammation represents an important avenue of medical intervention to improve patient outcomes [4]. Recent findings implicate Eph receptor signaling in the pathophysiology of neurological disorders [5–7]. The Eph receptors comprise the largest family of receptor tyrosine kinases that are subdivided into two classes, EphAs and EphBs [8]. EphAs contains a single transmembrane fragment and several cytoplasmic domains, while EphBs are GPI-anchored proteins. The extracellular portion of the Eph receptor interacts with its ligands ephrins, and their binding induces bi-directional signaling which has been implicated in multiple physiological and developmental processes. Ephrin type-A receptor 4 (EphA4) has been implicated in the disease pathology of Alzheimer’s [9], amyotrophic lateral sclerosis [10], ischemia [11], and in TBI [12]. Our initial findings of neuroprotection in global EphA4^−/−^ mice suggest EphA4 contributes to neural tissue damage. However, recent findings demonstrate neuron-specific Emx1-Cre conditional EphA4 knockout mice did not demonstrate neuroprotection following TBI, suggesting EphA4 mediates injury through non-neuronal mechanism(s) [13]. Interestingly, previous studies have implicated EphA4 in promoting the adhesion of monocytes to endothelial cells within atherosclerotic plaques [14] and mediating CD4(+) T cell development [15] and migration [16–18]. However, the mechanistic role of EphA4 signaling in regulating cell type-specific peripheral immune response to tissue damage remains unknown.

The current study evaluated the role of EphA4 in regulating inflammation and neural tissue damage following TBI using bone marrow chimeric knockout mice. We further tested the systemic delivery of two known EphA4 blocking peptides KYLPYWPVLSSL (KYL) [19] and VTMEAINLAFPGEEKK (VTM-EEKK) which shows high binding affinity to EphA4 [20]. Our findings demonstrate pharmacological inhibition and gene-targeted deletion of hematopoietic-specific EphA4 provided neuroprotection by modulating the pro-inflammatory milieu induced by the peripheral immune system following TBI. Additional in vitro analysis suggests these effects may be regulated by EphA4 suppression of monocyte/macrophages anti-inflammatory polarization state potentially through the mTOR, p-Akt, and NF-κB pathways. The current findings highlight a new and novel role for a well-characterized central nervous system axon guidance molecule in the acute immune response to TBI, which may be applicable to another disease of the nervous system.

## Methods

### Animals

All mice were housed in an AAALAC-accredited, virus/specific antigen-free facility with a 12-h light-dark cycle and food and water ad libitum. All mice used in these studies were male mice in order to reduce variables with sex differences. CD1 mice were purchased from Charles Rivers and reared until age P60–P90 for experimentation. *Cx3Cr1*^GFP/+^, *Epha4*^−/−^, *Epha4* floxed, Rosa^mTmG^, and *Tie2*-Cre mice on the C57BL/6 background were purchased from the Jackson Labs (Jackson Laboratory, Bar Harbor, ME) and bred for experimentation to a CD1 background and genotyped as previously described [11]. All experiments were conducted in accordance with the NIH Guide for the Care and Use of Laboratory Animals and were conducted under the approval of the Virginia Tech Institutional Animal Care and Use Committee (IACUC; #15-063) and the Virginia-Maryland College of Veterinary Medicine.

### Adoptive transfer

Wild-type *EphA4*^*f/f*^ male mice were X-ray irradiated with two doses of 550 rad at least 6 h apart to ablate the bone marrow. Mice were placed on autoclaved and filtered 1 mg/ml gentamycin sulfate water for 3 days prior and 2 weeks following irradiation. Donor *Tie2-Cre*^*mtmg*^ and *EphA4*^*f/f*^*/Tie2-Cre*^*mtmg*^ male mice were euthanized, and the bone marrow was flushed into FBS-containing media with penicillin-streptomycin. Red blood cells were lysed, and bone marrow cells were resuspended in sterile PBS. Irradiated mice were reconstituted with one to five million BMCs via tail vein injection within 24 h of irradiation then controlled cortical impact (CCI) injury was performed 28 days post-injection.

### Bead isolation of CD45+ immune cells

Male mice were euthanized, and CD45+ cells were isolated from the lesion area as previously described [21]. Briefly, the brains were placed in L15 dissecting media (Thermo Fisher, Waltham, MA) before the 4 × 4 mm lesion area was dissected and neural dissociation was performed (kit from Miltenyi Biotech, Auburn, CA). Seven mice were pooled per group (WT^WTBMC^ and WT^KOBMC^), and a single-cell suspension was prepared. The suspension was subjected to CD45+ magnetic microbeads and column separation (MACS; Miltenyi Biotech, Auburn, CA). The flow-through was collected. The CD45+ and final flow-through fractions were placed in Trizol and used for RNA isolation and qPCR. Technical triplicates of the pooled samples were used for qPCR.

### Peptide sequences

Three peptide sequences were synthesized: VTM-EEKK (VTMEAINLAFPGEEKK), VTA-EEKK (VTAEAINLAFPGEEKK), and KYL (KYLPYWPVLSSL). All peptides were synthesized via solid-phase peptide synthesis using Rink amide MBHA resin. Amino acids and resin were purchased from P3BioSystems. *N*,*N*-Diisopropylethylamine (DIEA), 1,8-diazabicyclo [5.4.0] undec-7-ene (DBU), triisopropylsilane (TIPS), and 2-(1*H*-benzotriazol-1-yl)-1,1,3,3-tetramethyluronium hexafluorophosphate (HBTU), and all other reagents were purchased from commercial vendors and used as received. A preparative RP-HPLC (Agilent Technologies 1260 Infinity) with an Agilent PLRP-S column (10 μm, 100 Å) was used to purify peptides. Fractions after HPLC purification were checked by an ESI-MS (Advion Express CMS), and then product-containing fractions were dried by a lyophilizer (LabConco FreeZone 6Plus). The final products were analyzed by a matrix-assisted laser desorption ionization tandem time of flight mass spectrometer (4800 MALDI TOF/TOF; AB Sciex).

### Preparation of VTM-EEKK, VTA-EEKK, and KYL peptides

Due to poor hydrosolubility of the VTM peptide, we modified the sequence by adding four hydrophilic amino acids (EEKK) to its C-terminus (VTMEAINLAFPGEEKK; VTM-EEKK). The VTM-EEKK and VTA-EEKK control peptides were synthesized manually via solid-phase peptide synthesis (SPPS) in a shaker vessel using standard Fmoc protocols. The coupling, deprotection, and cleavage solutions were prepared following published methods. An example synthesis of VTM-EEKK is as follows: Rink amide MBHA resin (1 equiv., 0.25 mmol) was added to the shaker vessel and swollen for 15–20 min in 15 ml DMF. The Fmoc group was then deprotected using DBU/piperidine in DMF, and Fmoc-Lys (Boc)-OH (4 equiv., 0.47 g) was coupled using HBTU and DIEA in DMF for 3 h. Coupling was confirmed by the lack of blue color in a Kaiser test. The VTM-EEKK peptide was cleaved by adding 15 ml of cleavage solution (2.5% H_2_O, 2.5% TIPS in H_2_O) to the shaker vessel and then shaking for 2.5 h. The peptide solution was drained and collected in a round-bottom flask and then concentrated via rotary evaporation until less than 1 ml solution was left. The peptide was precipitated by pouring cold ethyl ether into the round-bottom flask, and white precipitated peptide powder was recovered by filtration. The crude peptide powders were purified via preparative RP-HPLC, eluting on an Agilent PLRP-S column with H_2_O and ACN as mobile phases, with 0.1% NH_4_OH added to each. Pure, product-containing fractions were collected after HPLC and checked by ESI-MS then lyophilized. Aliquots (3 mg each) were prepared by dissolving pure peptide at 10 mg ml^−1^, adjusting to pH 7 using 0.1 mg ml^−1^ NaOH, and transferring 300 μl into microcentrifuge tubes. The aliquots were lyophilized and then stored at − 20 °C before use.

### Controlled cortical impact

Male mice were anesthetized with ketamine (100 mg/kg) and xylazine (10 mg/kg) via intraperitoneal injection and positioned in a stereotaxic frame. Body temperature was monitored with a rectal probe and maintained at 37 °C with a controlled heating pad set. The *Φ* = 4 mm craniotomy was made using a portable drill over the right parietal-temporal cortex (− 2.5 mm A/P and 2.0 mm lateral from the bregma). The injury was induced by a program-controlled cortical impactor (*Φ* = 3-mm beveled tip) connected to an eCCI-6.3 device (Custom Design & Fabrication, LLC) at a velocity of 5.0 m/s, depth of 2.0 mm, and 100 ms impact duration. Following injury, the incision was closed using Vetbond tissue adhesive (3M, St. Paul, MN, USA), and the animals were placed into a heated cage and monitored every 20 min until fully recovered from anesthesia. Alzet® Mini-Osmotic Pumps Model 1007D (Catalog # 0000290, DURECT Corporation, Cupertino, CA) was used to provide continuous systematic delivery of the saline control, VTA-EEKK, VTM-EEKK, or KYL peptide. The dosage of each peptide administration was 10 mg/kg/day.

### Blood-brain barrier analysis

BBB disruption following CCI injury was performed as previously described [21]. Briefly, a 2% sterile Evans blue (EB, Sigma E2129) solution was prepared in 0.1 M PBS and passed through a 0.22-μm filter. Mice having undergone either sham or CCI injury were restrained and injected with 5 μl g^−1^ EB solution via the tail vein. Three hours post-injection, the brains were removed and the ipsilateral and contralateral cortical hemispheres dissected and incubated separately in 500 μl formamide (Invitrogen, 15515-026) for 24 h at 55 °C. Samples were then centrifuged to pellet the tissue, and absorbance of the solution was measured at 610 nm using a NanoDrop 1000 Spectrophotometer (Thermo Scientific, Wilmington, DE.). Absorbance at 610 nm was quantified and graphed for each cortical hemisphere.

### Evaluation of lesion volume

Lesion volume (mm^3^) was assessed by a blinded investigator using Cavalieri Estimator from StereoInvestigator (MicroBrightField, Williston, VT, USA) and an upright Olympus BX51TRF motorized microscope (Olympus America, Center Valley, PA, USA) as previously described [5, 22]. Briefly, a volume analysis was performed by estimating the area of tissue loss in the ipsilateral cortical hemisphere using five 30 μm serial coronal sections (− 1.1 to − 2.6 mm posterior from bregma). Nissl-stained coronal sections were viewed under fluorescent microscopy at a magnification of × 4. A random sampling scheme was used that estimates every tenth section from rostral to caudal, yielding five total sections to be analyzed. A randomly placed grid with 100-μm spaced points was placed over the ipsilateral hemisphere, and the area of contusion was marked within each grid. Lesion boundaries were identified by loss of Nissl staining, pyknotic neurons, and tissue hemorrhage. The marked areas, using grid spacing, were then used to estimate total tissue volume based on section thickness, section interval, and total number of sections within the Cavalieri probe, StereoInvestigator. Data are represented as the volume of tissue loss or damage (mm^3^) for juvenile and adult mice.

### Endothelial cell growth and LPS stimulation assays

Endothelial cells were isolated from postnatal day 1–3 brains of EphA4^f/f^ (WT) and EphA4^f/f^ /Tie2-Cre then grown in Cell Biologics™ Complete Mouse Endothelial Cell Medium (Catalog # M1168, Chicago, IL) as previously described [11]. To simulate the endothelial cell response to inflammation, we plated 300,000 cells/well in a 6-well dish in complete media overnight. The following day, we added 1 μg ml *Escherichia coli* O111:B4 LPS (Sigma Aldrich, St. Louis, MO) in the presence or absence of KYL (500 μM) and VTM (500 μM) peptides. Cells were washed two times with cold sterile PBS prior to RNA isolation and subsequent analyses. Concentrations used were determined through dosage studies.

### Western blot

Cells were washed 3× with cold 1× PBS, or freshly dissected cortices were lysed in RIPA buffer (Tris-base 50 mM, NaCl 150 mM, EDTA 1 mM, NP-40 1%, sodium deoxycholate 0.25%, NaF 20 mM, 1 mM Na_3_VO_4_ 1 mM, β-glycerophosphate 10 mM, azide 0.02%) with Roche Proteinase Inhibitor Cocktail (Catalog # 25178600, Indianapolis, IN) and Thermo Fisher Scientific Pierce™ Phosphatase Inhibitors (Catalog # 88667, Waltham, MA). The total amount of protein was quantified by Lowry method (DC Protein Assay Kit, catalog # 500-0116, Bio-Rad, Hercules, CA). Then, 50 μg total protein of each sample was separated by 8% SDS-PAGE then blotted on to Bio-Rad Laboratories Immin-Blot™ PVDF membrane (Catalog # 162-0177, Hercules, CA). The membranes were incubated with primary antibodies in blocking solution: TBS/0.1% Tween20 (TBST)/5% bovine serum albumin (BSA) for overnight at 4 °C, washed 4× with TBST, and incubated with secondary antibodies (anti-rabbit IgG Dylight™ conjugate 680 or anti-mouse IgG Dylight™ conjugate 800; Cell Signaling Technology, Danvers, MA) for 2 h in blocking solution at room temperature (Table 1). Following 4× wash with TBST, images were acquired by using LI-COR Odyssey Imaging Systems (LI-COR, Inc.), and band intensities were quantified by using NIH ImageJ software.

**Table 1:** Information of antibodies

Antibody	Provider	Catalog number	Dilution
β-actin	Cell Signaling Technology	3700S	1:10,000
Akt	Cell Signaling Technology	4691P	1:3000
p-Akt (S473)	Cell Signaling Technology	4060S	1:3000
BrdU	Abcam (IHC)	Ab6326	1:500
Cx43	ECM Biosciences	CM4961	1:3000
EphA4	ThermoFisher (IHC)	371,600	1:250
p-Cx43 (S368)	Cell Signaling Technology	3511S	1:3000
EphA4	ECM Biosciences (Western)	EM2801	1:3000
ERK	Cell Signaling Technology	9102S	1:3000
p-ERK (T202/Y204)	Cell Signaling Technology	9101S	1:3000
DyLight680-conjugated anti-rabbit IgG (H+L)	Cell Signaling Technology	5366S	1:10,000
DyLight800 conjugated anti-mouse IgG (H+L)	Cell Signaling Technology	5257S	1:10,000

### Immunohistochemistry and confocal image analysis

The freshly dissected whole brain was snap-frozen and cryosectioned in serial 30-μm sections. Sections were fixed with 10% buffered formalin, washed 3 times in 1× PBS, and blocked in 2% cold water fish gelatin (Sigma, Inc.) in 0.2% triton for 1 h. The sections were then exposed to mouse anti-EphA4 (ThermoFisher, Cat #:371600) antibody (1:100) in block overnight, washed with 1× PBS then treated with anti-mouse alexFluor594 for 1 h. The sections were further washed in 1× PBS then mounted in media with DAPI counterstain (SouthernBiotech). Images were acquired using a Zeiss 880 confocal microscope (Carl-Zeiss, Oberkochen, Germany).

### Quantitative real-time PCR

Total RNA from 4 × 4 mm ipsilateral sham- or CCI-injured cortical tissue was isolated according to the manufacturer’s instructions using TRIzol® reagent (Ambion), and total RNA was isolated from the blood using TRIzol® Reagent LS per manufacturer’s instructions. RNA quantification was carried out by measuring absorbance with spectrophotometer ND-1000 (NanoDrop). RNA was reverse transcribed into cDNA with iScript™ cDNA synthesis kit (Biorad, Hercules, CA) per manufacturer’s specifications. For qRT-PCR analysis, 50 ng cDNA per reaction was amplified using iTaq™ Universal SYBR® Green Supermix (Biorad, Hercules, CA). Expression changes were calculated using ΔCq values with reference to *β-actin* internal control gene for cultured cells and *Gapdh* internal control gene for all other samples. Relative expression was calculated then normalized and compared to appropriate sham or untreated samples. All primers were tested for primer efficiency which ranged from 87 to 113% (Table 2).

**Table 2:** Information of qPCR primers

Gene	Primer sequence (5′-3′)
*β-actin*	Fw: TCGTACCACAGGCATTGTGATGGA
	Rv: TGATGTCACGCACGATTTCCCTCT
*Angpt2*	Fw: GGAAAAGCAGATTTTGGATCAG
	Rv: TTCTGCTCCTTCATGGACTGTA
*Arg1*	Fw: AAGATAGGCCTCCCAGAACCG
	Rv: AAAGGCCGATTCACCTGAGC
*Ccr2*	Fw: GGGCTGTGAGGCTCATCTTT
	Rv: TGCATGGCCTGGTCTAAGTG
*Gapdh*	Fw: CGTCCCGTAGACAAAATGGT
	Rv: TCAATGAAGGGGTCGTTGAT
*Il12p40*	Fw: AGACCCTGCCCATTGAACTG
	Rv: GAAGCTGGTGCTGTAGTTCTCATATT
*Il6*	Fw: CTTCACAAGTCGGAGGCTTAAT
	Rv: GATTGTTTTCTGCAAGTGCATC
*Kc (cxcl1)*	Fw: ACCCAAACCGAAGTCATAGCCACA
	Rv: AGTGTTGTCAGAAGCCAGCGTTCA
*Mcp1*	Fw: TCACCTGCTGCTACTCATTCACCA
	Rv: TACAGCTTCTTTGGGACACCTGCT
*Tnf*	Fw: AGAAGAGGCACTCCCCCAAA
	Rv: TGAGGGTCTGGGCCATAGAA
*Tie2*	Fw: AAATGACCCTAGTGAAGCCAGA
	Rv: GTCAGGAGGTAAGACTCGGTTG
*Epha4*	Fw: AAAAATGTACTGTGGGGCAGAT
	Rv: TCCGTGGAAAGAGCTTTGTAAT
*Vcam*	Fw: GGTGTACGAGCCATCCACAG
	Rv: ACTTGTGGAAATGTGCCCGA
*Gja1 (cx43)*	Fw: CGGAAGCACCATCTCCAACT
	Rv: CCACGATAGCTAAGGGCTGG
*Il6r*	Fw: CTGTTTGCAACGCACAGTGA
	Rv: AACACCACCAACGGGAAGAG
*Cd86*	Fw: TGTGCCCAAATAGTGCTCGT
	Rv: TCTGCCGTGCCCATTTACAA

### Macrophage culture

Bone marrow cells (BMCs) isolated from 8- to 12-week WT CD1 background mice were cultured in DMEM medium supplemented with 10% fetal bovine serum, 2 mM l-glutamine, 1% penicillin/streptomycin, and 10 ng ml^−1^ M-CSF. Briefly, bone marrow-derived macrophages (BMDMs) were isolated from the femurs, filtered through a 70-μm filter; red blood cells were lysed using ACK lysing buffer (Gibco); and cells were cultured at 1 × 10^6^ cells ml^−1^ in complete DMEM medium. Cells received fresh media containing 10% FBS, 2 mM l-glutamine, 1% penicillin/streptomycin, and 10 ng ml^−1^ M-CSF on days 2 and 4. After 5 days, cultured cells were washed with PBS and given fresh DMEM containing no M-CSF or FBS for subsequent treatments with VTM and KYL peptide. BMDMs were allowed to equilibrate for 2 h in fresh DMEM medium prior to VTM and KYL peptide treatment. The BMDMs were treated with KYL (500 μM) and VTM (500 μM) 1 h prior to 4 h treatment with 1 μg ml^−1^ *Escherichia coli* O111:B4 LPS (Sigma Aldrich, St. Louis, MO). Polarization studies were performed by changing media on day 5 to DMEM containing 10% FBS, 2 mM l-glutamine, 1% penicillin/streptomycin, and 5 ng ml^−1^ M-CSF. Day 5 BMDMs were then treated with IL-4 (20 ng/ml; R&D systems) or IFNγ (80 ng/ml; R&D Systems) for 48 h for M2 or M1 polarization, respectively. Cells were washed two times with cold sterile PBS prior to RNA isolations and subsequent analyses. All concentrations were determined through dosage studies.

### Phospho microarray

Using a commercially available high-throughput ELISA-based antibody Cancer Signaling Phosphoarray (Full Moon Biosystems, Inc., Sunnyvale, CA), we analyzed the total and phospho-protein changes between WT and EphA4^−/−^ BMDMs. BMDMs were cultured as described previously and treated with PBS or 1 μg ml^−1^ *Escherichia coli* O111:B4 LPS (Sigma Aldrich, St. Louis, MO) for 4 h prior to protein isolation. Cells were washed 3× with cold 1× PBS and lysed in RIPA buffer (Tris-base 50 mM, NaCl 150 mM, EDTA 1 mM, NP-40 1%, sodium deoxycholate 0.25%, NaF 20 mM, 1 mM Na_3_VO_4_ 1 mM, β-glycerophosphate 10 mM, azide 0.02%) with Roche Proteinase Inhibitor Cocktail (Catalog # 25178600, Indianapolis, IN) and Thermo Fisher Scientific Pierce™ Phosphatase Inhibitors (Catalog # 88667, Waltham, MA). The total amount of protein was quantified by the Lowry method (DC Protein Assay Kit, catalog # 500-0116, Bio-Rad, Hercules, CA). Protein was purified using the buffer exchange/lysate purification system given with the Cancer Signaling Phospho microarray. Then 100 μg of purified protein for each sample was used for the remaining of the protocol per manufacturer’s instructions. GenePix Microarray Scanner, 4000B (Molecular Devices, LLC., San Jose, CA) was used to image the microarray, and GenePix Pro (Molecular Devices, LLC., San Jose, CA) software was used for subsequent analyses. Full Moon Biosystems analyzed the data using the average signal intensity of six individual blots, for each pair of site-specific antibody and phosphosite-specific antibody; signal ratio of the paired antibodies was determined. A fold change was considered significant when the value was less than 0.5 or greater than 1.5 (Additional file 1). A 95% CI was used to quantify the precision of the phosphorylation ratio based on the analysis of the six individual sample replicates.

### Statistical analysis

Data was graphed using GraphPad Prism, version 7 (GraphPad Software, Inc., San Diego, CA). Student’s two-tailed *t* test was used for comparison of the two experimental groups. For three or more groups, multiple comparisons were done using one-way and two-way ANOVA where appropriate followed by Bonferroni post hoc test for multiple pairwise examinations. Changes were identified as significant if *p* was less than 0.05. Mean values were reported together with the standard error of the mean (SEM). The sample size was determined based on an effect size measured for each outcome by pilot or prior studies. G*Power 3 (Universitat Dusseldorf, Germany) was used to retrieve sample size using an acceptable power range between 80 and 90%. All animal and serial sections were coded, and a double-blinded strategy was used in all stereological analysis.

## Results

### Meta-analysis of ephA4 gene expression and protein changes following CCI injury

**Fig. 1:**
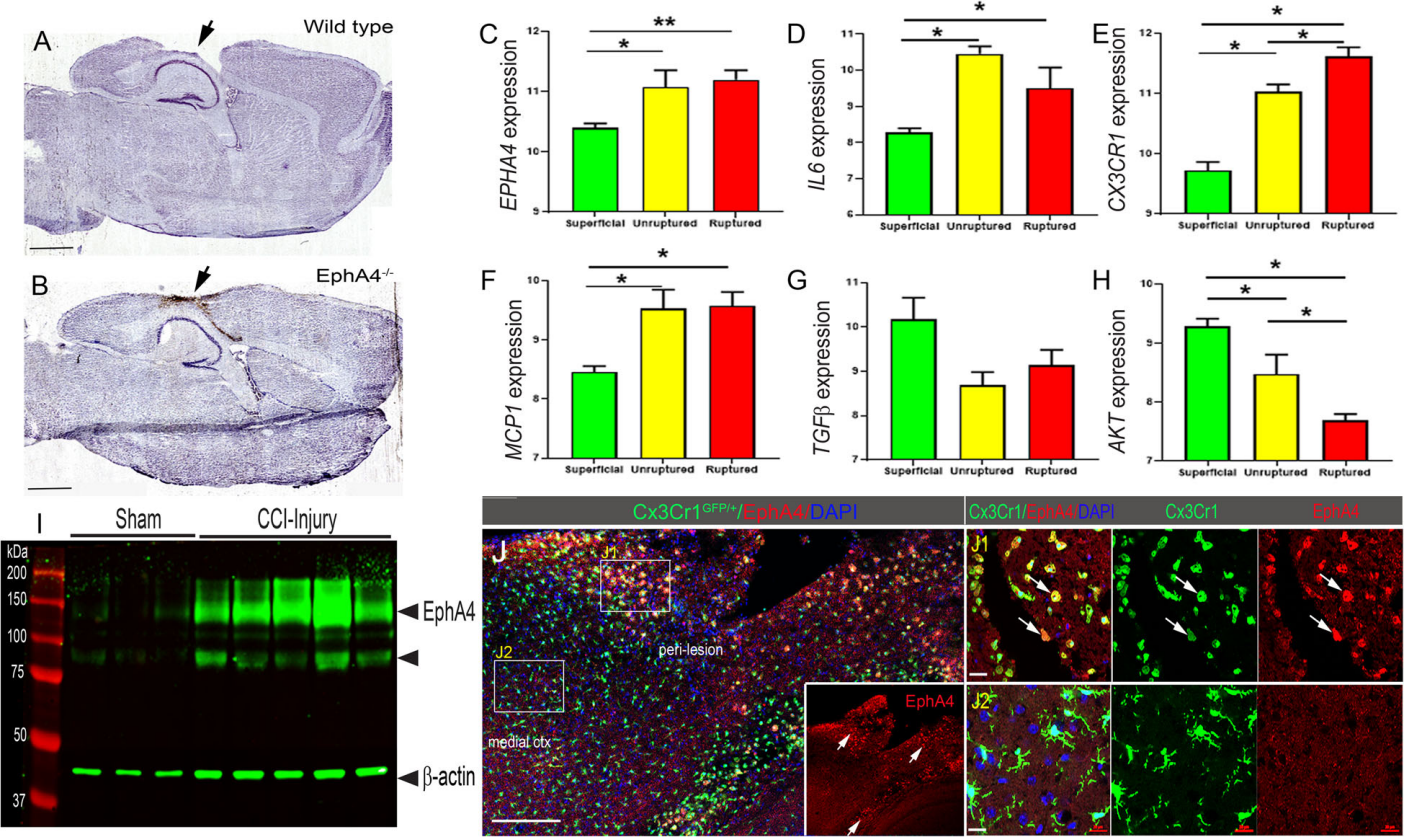
Neuroprotection in EphA4^−/−^ mice and human meta-analysis data of EPHA4. **a**, **b** Nissl staining of sagittal sections from WT or EphA4 global knockout mice at 14 days post-CCI injury compared to wild type. **c**–**h** Retrospective analysis of data archived as NIH GEO datasets from human patients following brain aneurysms. This study evaluated the gene expression on > 41,000 transcripts. Superficial, *n* = 10; unruptured, *n* = 5; ruptured, *n* = 8. **i** Western blot analysis for EphA4 protein expression in CCI-injured cortical tissue at 2 h compared to sham-injured cortices (*n* = 3–5/group). **j** Representative confocal images of anti-EphA4 immuno-labeling (red; inset) at 24 h post-CCI injury in the ipsilateral cortex of CX3CR1^GFP/+^ mice. CX3CR1-expressing microglia and/or infiltrating monocytes/macrophages show high expression of EphA4 in the peri-lesion cortex (J1) compared to adjacent cells in the medial parietal cortex (J2). Scale bar in **j** = 200 μm; scale bar in (J1) and (J2) = 20 μm. ANOVA with Bonferroni post hoc test. **p < 0.01, ***p < 0.001 compared to superficial aneurysms

Our initial findings show cortical neuroprotection in global *EphA4*^−/−^ mice (Fig. 1b) compared to wild-type mice (Fig. 1a) at 14 days following controlled cortical impact (CCI) injury. This represents a sub-chronic effect on lesion volume. Next, we observed the earliest changes in the expression of EphA4 in the acute phase of injury, at 2 h post-CCI injury. At this time, we found a significant increase in EphA4 protein levels in the ipsilateral CCI-injured murine cortex of wild-type mice compared to ipsilateral sham (Fig. 1i). Next, we sought to observe major changes in EphA4 expression in the injured cortex using immunohistochemistry. In order to perform this, we utilized the *Cx3cr1*^*GFP/+*^ knockin mice to evaluate the cell type-specific expression of EphA4 on *Cx3cr1*-positive cells in the damaged cortex after CCI injury. Confocal image analysis demonstrates EphA4 is expressed throughout the peri-lesion cortex on *Cx3cr1*^GFP^-positive cells (Fig. 1j, J1 inset) but not on *Cx3cr1*-expressing resident microglia medial to the lesion site; therefore, we focused our studies on the infiltrating monocytes in further experiments (Fig. 1j, J2). Next, we comparatively assessed the *EPHA4* expression in human tissue using a data mining bioinformatics approach. We conducted a retrospective analysis of gene expression data archived as NIH GEO datasets from human patients following ruptured brain aneurysms (GSE26969; GSE54083) [23] since datasets for TBI are not freely available. Much like TBI, ruptured brain aneurysms also induce an influx of peripheral immune cell infiltration into the brain, and increases in IL1β, IL-6, MCP1, and TNF have been associated with poor outcomes for both [24, 25]. Infiltrating monocytes also express similar patterns of MMPs 2 and 9 in both the TBI and aneurysms which has a substantial impact on the injured milieu [26–28]. We conducted a metadata analysis on publically available GEO datasets from an independent study evaluating the gene expression changes in specimens collected from the aneurysmal dome following either superficial, unruptured, or ruptured intracranial aneurysms [29]. We find significant increases in *EPHA4* expression concomitant with *IL6*, *CX3CR1*, and *MCP1* in unruptured and ruptured aneurysms compared to superficial (Fig. 1c–f). Conversely, *TGFβ* and *AKT* were decreased with *AKT* being significant (Fig. 1g and h, respectively). These findings illustrate EphA4 may play a substantial role in regulating immune-derived signals induced following TBI.

### Peptide inhibition of EphA4 reduces cortical lesion volume and attenuates inflammatory gene expression after TBI

**Fig. 2:**
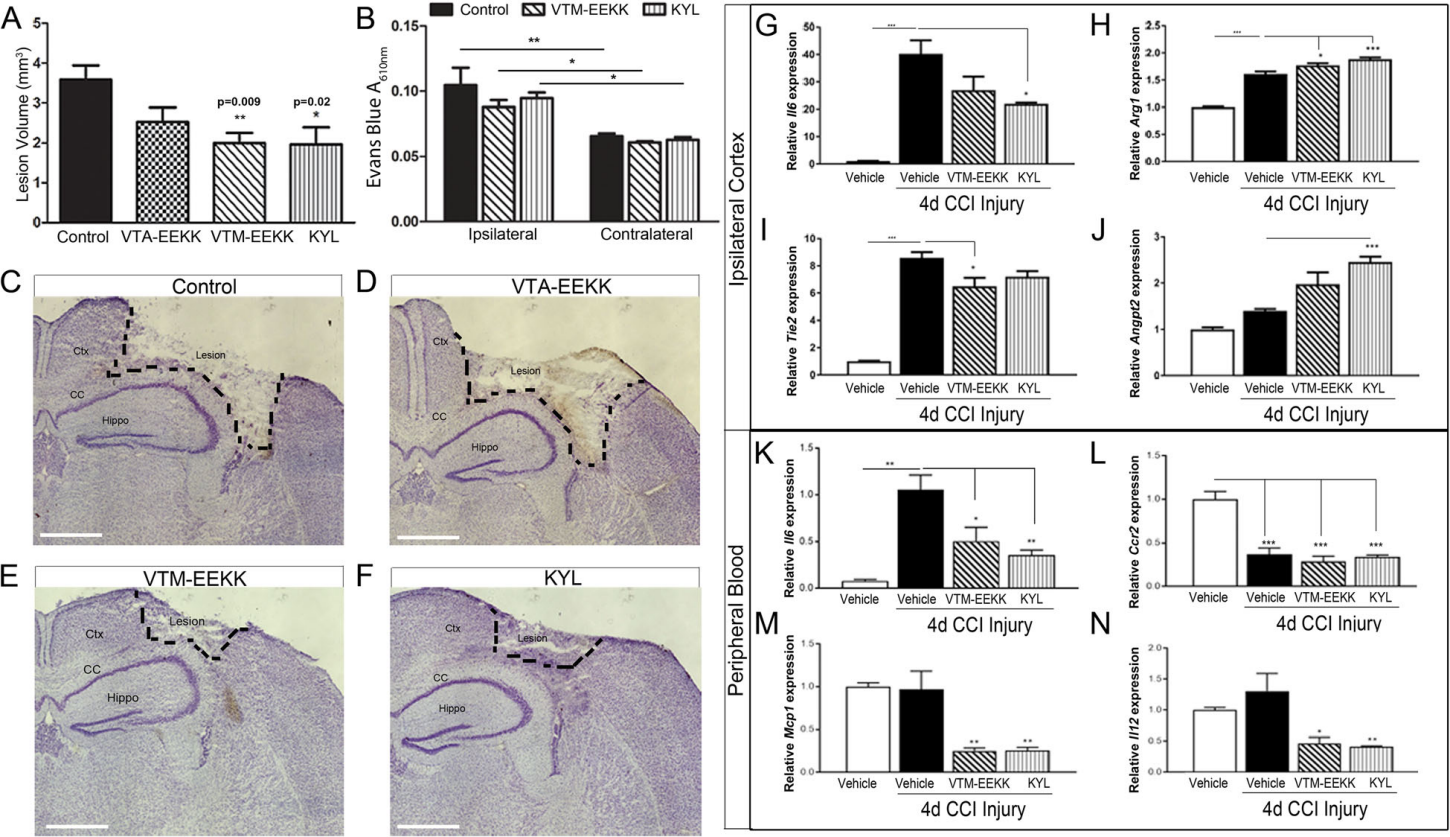
EphA4 blocking peptides provide neuroprotection and reduce the pro-inflammatory response following 4 days CCI injury. **a** Quantified lesion volume at 4 days post-CCI injury in mice implanted with vehicle control, VTA-EEKK control, VTM-EEKK, and KYL peptides. *p < 0.05 compared to vehicle. **b** BBB disruption as measured by Evans blue absorbance (610 nm). Whole cortex EB absorbance is compared between and within the ipsilateral or contralateral hemisphere between vehicle, VTM-EEKK, and KYL-treated mice. *p < 0.05, **p < 0.01 compared to contralateral. **c**–**f** Representative images of Nissl-stained ipsilateral cortex at 4 days post-CCI injury in mice infused with vehicle, VTA-EEKK, VTM-EEKK, and KYL. **g**–**j** Quantified mRNA expression of pro-inflammatory *Il6* and pro-resolving *Arg1*, *Tie2*, and *Angpt2* respectively in the ipsilateral cortex relative to vehicle-infused sham injury vs vehicle 4 days post-CCI and after KYL and VTM-EEKK treatment. **k**–**n** Quantified mRNA expression in the whole blood of *Il6*, *Ccr2*, *Mcp-1*, and *Il12* respectively from vehicle sham vs CCI-injured vehicle, KYL, VTM-EEKKK-infused mice. *p < 0.05, **p < 0.01, ***p < 0.001. *n* = 6 per group. Scale bar = 1 mm. Ctx, cortex; CC, corpus callosum; Hippo, hippocampus. *n* = 5–8 per group

To determine the acute effects of systemic EphA4 inhibition on TBI outcome, we utilized mini-osmotic pumps implanted subcutaneously (s.q.) containing either KYL, VTM-EEKK, VTA-EEKK control peptides, or vehicle alone to provide continuous systemic delivery at 0.5 μl/h immediately following injury for 4 days post-CCI. We chose to investigate 4 days post-injury in order to allow a clinical application of a systemic delivery of EphA4 peptide inhibitor during the time infiltrating monocytes are most prevalent (days 1–4). Control mice receiving vehicle alone displayed 3.58 ± 0.36 mm^2^ cortical lesion volume (Fig. 2a, c). Significant neuroprotection was seen in mice administered with EphA4 antagonistic peptides, VTM-EEKK (2.00 ± 0.26 mm^2^; *p* = 0.011) (Fig. 2a, e), and KYL (1.97 ± 0.43 mm^2^; *p* = 0.024) (Fig. 2a, f) but not following VTA-EEKK control peptide infusion (2.53 ± 0.36 mm^2^; *p* = 0.323) (Fig. 2a, d). We found significant BBB disruption in the ipsilateral cortex compared to contralateral at 4 days post-CCI injury, which was not influenced by EphA4 inhibition (Fig. 2b; *f* (2, 10) = 1.041, *p* = 0.388).

Next, we analyzed mRNA from the ipsilateral injured cortex and whole peripheral blood at 4 days post-sham or post-CCI injury. This time point represents the sub-acute phase of injury where both pro-inflammation and pro-resolving cues are expressed. We found a 40-fold increase in *Il6* mRNA expression in the injured cortex compared to sham. Interestingly, CCI-injured mice infused with KYL showed a significant reduction in *Il6* (*p* = 0.033), while VTM-EEKK showed a trend towards reduced expression (Fig. 2g, *p* = 0.1508). Moreover, we found a concomitant increase in pro-resolving arginase-1 (*Arg1*) (Fig. 2h; VTM-EEKK *p* = 0.036 and KYL *p* = 0.0009), angiopoietin-2 (*Angpt2*) (Fig. 2j; KYL *p* = 0.0009), and reduced *Tie2* receptor, which is often downregulated as a feedback loop following overstimulation with angiopoietins [30] (Fig. 2i; VTM-EEKK *p* = 0.026) following VTM-EEKK and KYL treatment. Although 4 days post-CCI injury represents a sub-acute time point, we still observed a significant increase in the *Il6* expression in the whole peripheral blood, which was significantly attenuated with VTM-EEKK (*p* = 0.049) and KYL (*p* = 0.012) (Fig. 2k). We were unable to detect transcripts of *Angpt2* and *Tie2* at high enough levels in the peripheral blood and found no large changes in *Arg1*; therefore, we investigated other cytokines that may be upregulated in the blood. While we did not find CCI injury induces changes in the whole blood expression of monocyte chemoattractant protein-1 (*MCP1*) and *Il12p40* inflammatory genes at 4 days, we did find that VTM-EEKK (*p* = 0.009 and *p* = 0.019) and KYL treatment (*p* = 0.01 and *p* = 0.014) significantly reduced their homeostatic levels (Fig. 1m and n, respectively). While this time point may not fully demonstrate the full range of pro-inflammatory signaling changes following acute CCI injury, this data shows *Il6* remains a key inflammatory signaling in both the damaged cortex and peripheral immune compartment. Finally, TBI induced downregulation of *Ccr2* expression in the whole blood; however, no difference was found between CCI-injured vehicle and peptide inhibitor treatments (Fig. 2l), suggesting that inflammatory CCR2+ monocyte/macrophage populations may be depleted in the peripheral blood as they begin infiltrating the brain and that this process is not affected by systemic EphA4 inhibition. These findings, taken together, suggest EphA4 may mediate the pro-inflammatory milieu following TBI through regulation of the peripheral immune response.

### EphA4 bone marrow chimeric knockout mice show reduced lesion volume and pro-inflammatory gene expression

**Fig. 3:**
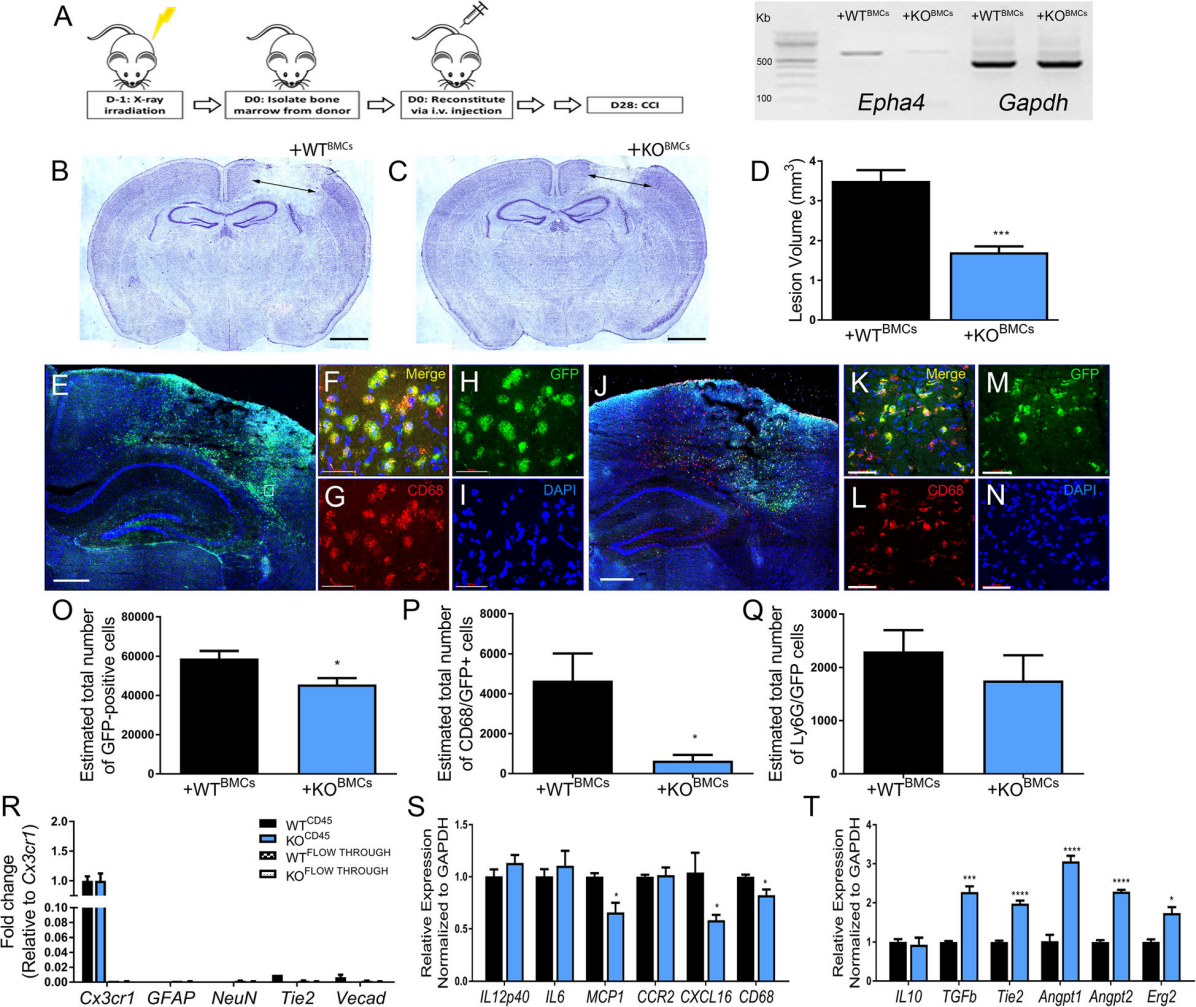
Bone marrow chimeric EphA4^−/−^ mice show decreased lesion volume and altered immune profile following CCI injury. **a** Bone marrow chimeric WT^WTBMC^ and WT^KOBMC^ mice were generated by irradiation and reconstitution with either wild-type or EphA4 knockout BMCs. PCR showing loss of transcript for Epha4 in the whole blood of WT^KOBMC^ mice compared to WT^WTBMC^. **b**–**d** WT^KOBMC^ mice display decreased lesion volumes compared to WT^WTBMC^ at 3 days following CCI injury. **e**–**i** Max projection of *z*-stack confocal images of the injured cortex showing GFP+ BMCS and DAPI (blue) from WT and **j**–**n** KO immune cell infiltration. **o** Non-biased stereological quantification showed reduced total GFP+ numbers and **p** co-labeled CD68/GFP-positive cells in the ipsilateral cortex of WT^KOBMC^ mice compared to WT^WTBMC^. **q** No difference was seen in the number of Ly6G/GFP+ neutrophils. **r** CD45-positive enriched cell isolation from the ipsilateral cortex at 3 days showed high purity of *cx3cr1* mRNA expression compared with *GFAP*, *Neun*, *Tie2*, and *Vecad*. **s** KO BMCs isolated from the injured cortex showed reduced pro-inflammatory *CD68*, *MCP1*, and *Cxcl16* expression concomitant with **t** increased pro-resolving *TGFβ*, *Tie2*, *Angpt1*, and *Angpt2*. *p < 0.05, ***p < 0.001, ****p < 0.0001 compared to the corresponding WT BMCs. **e** and **j** scale bar = 500 μm; **f**–**i** and **k**–**n** scale bar = 50 μm

Next, we sought to examine whether peripheral-derived EphA4 contributes to neural tissue damage following CCI injury. To test this, we utilized EphA4 chimeric knockout and wild-type mice (WT^KOBMCs^ and WT^WTBMCs^, respectively) where the loss of Epha4 transcript can be seen in the whole blood of WT^KOBMC^ mice (Fig. 3a). Chimeric animals were subjected to CCI injury, and lesion volume was analyzed using Nissl-stained serial coronal sections at 3 days following injury. Mice lacking EphA4 in the peripheral immune cell compartment showed reduced lesion volumes compared to mice with WT BMCs (Fig. 3b–d), indicating a neuroprotective effect in the absence of peripheral-derived EphA4. Donor BMCs from both wild-type and EphA4 knockout animals expressed GFP, allowing visualization and quantification of infiltrating peripheral-derived immune cells in the peri-lesion area. The peri-lesion cortex of WT^KOBMC^ mice displayed significantly less GFP+ peripheral immune cells (Fig. 3j–o) compared to WT^WTBMC^ (Fig. 3e–i, o). Moreover, the number of activated CD68+/GFP+ cells was significantly reduced in WT^KOBMC^ animals compared to WT^WTBMC^ (Fig. 3p). No difference in the number of Ly6G/GFP+ cells was observed between the groups (Fig. 3q). Isolation of CD45+ immune-derived/Cx3cr1-enriched cells (Fig. 3r) from the cortex using column bead separation showed reduced *Mcp1*, *Cxcl16*, and *Cd68* (Fig. 3s) and increased *Erg2*, *Tgfb*, *Tie2*, *Angpt1*, and *Angpt2* in the KO CD45+BMCs compared to WT (Fig. 3t). Interestingly, the loss of peripheral immune EphA4 did not attenuate the infiltrating immune cell expression of *Il6* suggesting KO BMCS either indirectly regulates *Il6* expression in the damaged ipsilateral cortex or the systemic EphA4 peptide inhibitors prevented *Il6* production on an alternative cell source in the damaged brain such as endothelial cells (see Additional file 2: Figure S2).

### EphA4 antagonists suppress the pro-inflammatory phenotype of LPS-stimulated monocyte/macrophages

**Fig. 4:**
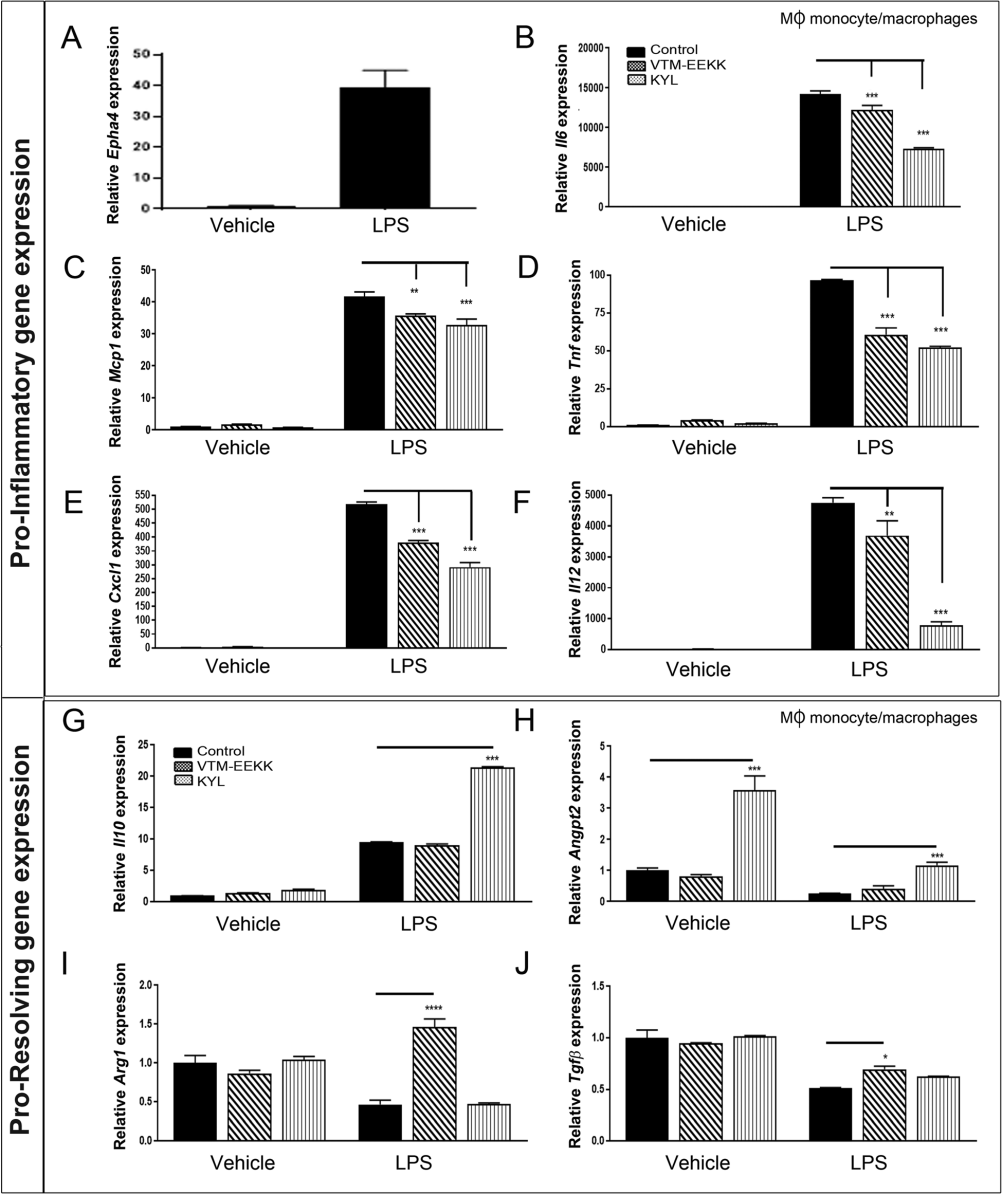
mRNA analysis of LPS-stimulated MΦ monocytes/macrophages treated with EphA4 peptide inhibitors. Quantified mRNA expression of pro-inflammatory genes *Epha4* (**a**), *Il6* (**b**), *Mcp1* (**c**), *TNF* (**d**), *Cxcl1* (**e**), and *Il12* (**f**) and of pro-resolving genes *Il10* (**g**), *Angpt2* (**h**), *Arg1* (**i**), and *Tgfβ* (**j**) following 4 h LPS stimulation of cultured MΦ monocytes/macrophages in the presence and absence of VTM-EEKK and KYL compared to vehicle control. *p < 0.05, **p < 0.01, ***p < 0.001, ****p < 0.0001 compared to the corresponding control PBS treatment. *n* = 3 biological replicates

Given that infiltrating monocyte/macrophages are the most prominent innate immune cell type present at this time [31, 32], are polarized towards several major subsets [33], and monocyte depletion confers neuroprotection after CCI injury [34], we further investigated the role of EphA4 on monocyte/macrophage inflammatory response. To test this, we induced a pro-inflammatory state in cultured bone marrow-derived MΦ monocyte/macrophages using 4-h lipopolysaccharide (LPS) stimulation and interrogated the gene expression profile in the presence and absence of EphA4 antagonists. LPS activates the TLR4 pathway similar to damage-associated molecular patterns (DAMPs) such as high mobility group box 1 (HMGB1), which are highly prominent post-TBI [35]. Following LPS stimulation, we found a significant 39-fold increase of EphA4 mRNA expression (Fig. 4a). Interestingly, we observed a significant decrease in mRNA levels of pro-inflammatory *Il6*, *Mcp1*, *Tnf*, *Cxcl1*, and *Il12* when monocytes were co-stimulated with LPS and EphA4 inhibitors VTM-EEKK or KYL compared to vehicle alone (Fig. 4b–f). Monocyte/macrophages show heterogeneous polarization in response to TBI and promote inflammation by releasing *Il12*, *Tnf*, *Il6*, and *Mcp1* following TBI [33]. On the other hand, we found monocyte/macrophages could be shifted from pro-inflammatory to pro-resolving following VTM-EEKK or KYL treatment. We observed increased expression of *Arg1*, a prominent anti-inflammatory macrophage marker [36]; anti-inflammatory *Il10*; A*ngpt2*, a pro-angiogenic stimulus that induces M2 marker expression in Tie2-expressing macrophages [37]; and *Tgfb* [38, 39] in the presence of EphA4 peptide inhibitors (Fig. 4g–j). Interestingly, we found divergent effects of VTM-EEKK compared to KYL on the pro-resolving gene expression of monocytes stimulated with LPS. These differences may represent a dose- or time-dependent effect in gene regulation in response to each inhibitor, which may not be fully evaluated at a single time point or dose. Nonetheless, these data implicate EphA4 in mediating pro-inflammation while suppressing an anti-inflammatory state in monocyte/macrophages.

Given that systemic delivery of EphA4 peptide inhibitors may influence the activation of EphA4 on other cell types in the brain after trauma, we further evaluated their potential effects on vascular inflammation by testing mRNA and protein expression following LPS stimulation in the presence and absence of VTM-EEKK and KYL treatment in primary cultured brain-derived endothelial cells [11]. Relative to vehicle-treated cells, we found *Il6*, *Cxcl1*, *Mcp1*, and *Cx43* were increased by LPS which was significantly reduced by KYL whereas only *Cxcl1* and *Mcp1* were suppressed by VTM-EEKK (Additional file 2: Figure S2A). The expression of vascular cell adhesion molecule (*VCAM*), which aides in leukocyte adhesion and recruitment [40], was also attenuated in the presence of VTM-EEKK and trended towards reduced expression in KYL. Similar to its effects in stimulated monocytes, we also found KYL increased *Angpt2* in KYL-treated cells. We also observed by western blot analysis that VTM-EEKK and KYL treatment had no effect on the total amount of EphA4 and p-ERK but significantly increased p-AKT expression (Additional file 2: Figure S2B-F). These findings demonstrate that EphA4 inhibition prevents pro-inflammatory gene expression induced in endothelial cells, which may also contribute to the cortical immune suppression, namely *Il6*, following therapeutic blockade of EphA4 in CCI injury.

### EphA4 regulates cultured monocyte/macrophage inflammatory state and Akt/NFkB signaling

**Fig. 5:**
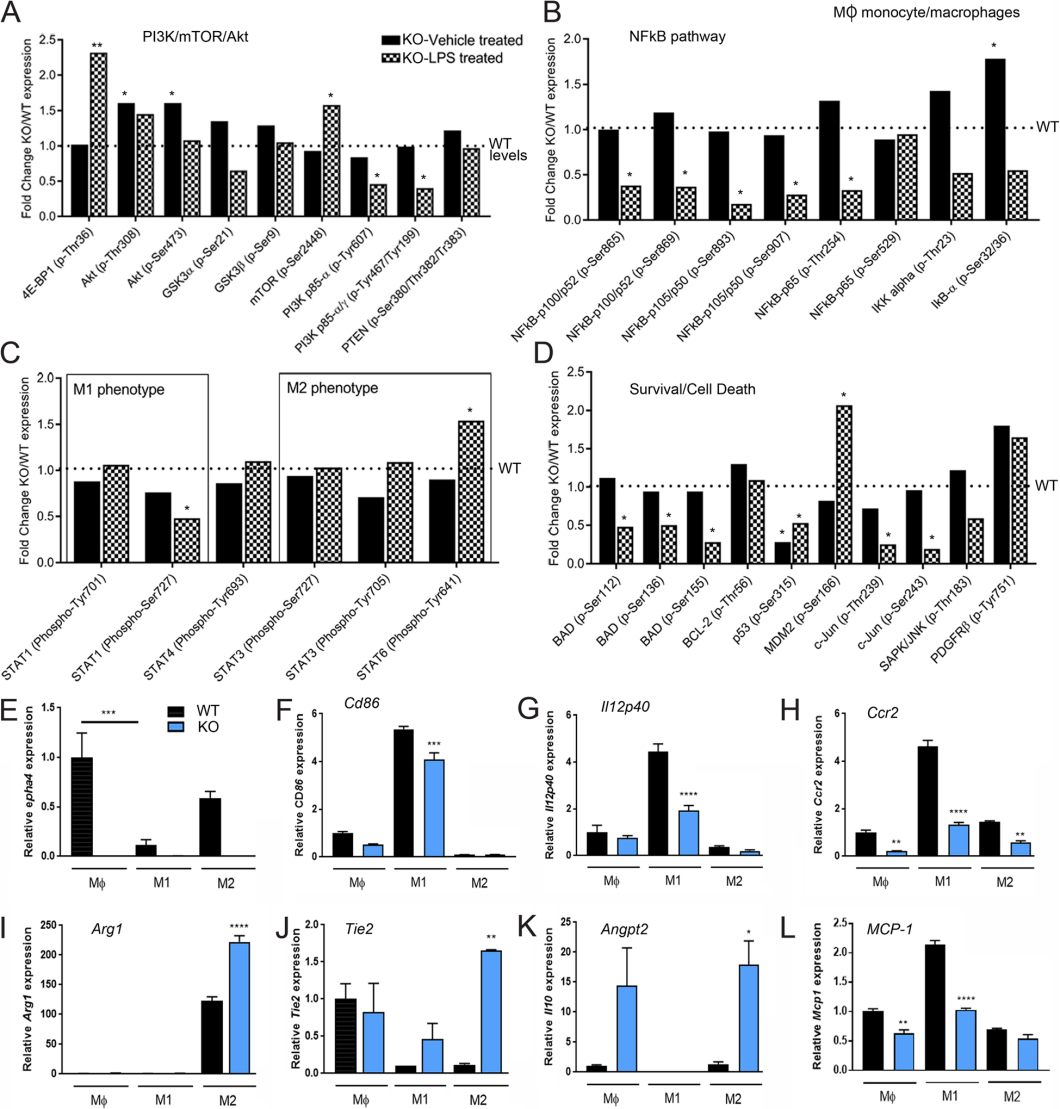
Phosphoarray analysis of LPS-stimulated wild-type and EphA4^−/−^ monocyte/macrophages. **a**–**d** Phospho(p) expression from 4 h LPS- vs vehicle-stimulated WT and KO MΦ monocytes using multiplex phosphoarray. Data is represented as fold change from average six replicates in vehicle KO cells (black bars) and LPS-treated KO cells (black checkered) compared to corresponding WT treated cells (dotted line). Any fold change above 1.5 and below 0.5 was considered significant with a 95% CI and was used to quantify the precision of the phosphorylation ratio based on the analysis of the replicates. **e**–**l** mRNA analysis in MΦ, M1, and M2 cells following polarization with PBS, IFNγ, and IL4, respectively. **e** Loss of *Epha4* transcript was confirmed in the KO cells. All M1/M2 data was normalized to WT MΦ levels. *p < 0.05, **p < 0.01, ***p < 0.001, ****p < 0.0001 compared to the corresponding control treatment

Akt plays a major role in regulating the anti-inflammatory phenotype of polarized monocyte/macrophages [41, 42], and we observed enhanced p-Akt expression in cultured ECs in the absence of Epha4. This led us to evaluate if p-Akt or other phosphorylated signaling molecules were dysregulated in WT and EphA4^−/−^ (KO) monocyte/macrophages following LPS stimulation. To test this, we used a commercially available high-throughput ELISA-based antibody phosphoarray. Company-generated results are highlighted in Additional file 1, showing fold change (KO/WT) as significant when the value was less than 0.5 or greater than 1.5. A 95% CI was used to quantify the precision of the phosphorylation ratio based on the average of six independent replicates per sample. Graphical data shows trending increased levels of Akt (p-Thr308) and significantly increased phosphorylated-4E-BP1 and mTOR (p-Ser2448) while reduced PI3Kp85-α (p-Tyr607) and PI3Kp85-α/γ (p-Tyr467/Tyr199) were observed in KO LPS-stimulated MΦ cells compared to WT levels (Fig. 5a). This correlated with reduced levels of phospho-NF-kB p100/p52, p105/p50, and p-65 (Fig. 5b) and M1-associated STAT1 p-Ser727 [43] while increased levels of M2-associated STAT6 p-Tyr641 [44] in KO compared to WT LPS-stimulated cells (Fig. 5b, c). STAT6 upregulation has been shown to promote anti-inflammatory behavior and is required for the expression of key anti-inflammatory modulator, Arginase-1 [45, 46]. Likewise, pathways involved in cell death and inflammation, namely BAD and JNK, showed reduced phosphorylation, while M2-associated MDM2/p53 axis [47] was positively altered in KO cells compared to WT (Fig. 5d). p53, which suppresses M2 genes, is ubiquitinated and degraded by MDM2. Interestingly, Akt can activate MDM2 [48]. Our results implicate Akt/NFkB/p53 as key downstream mediators of EphA4 signaling involved in suppressing the anti-inflammatory and pro-survival state of MΦ monocyte/macrophages.

Next, we tested the effects of genetic loss of EphA4 on the gene expression of polarized monocyte/macrophages in vitro. WT and KO monocytes were first polarized to M1 and M2 state utilizing IFNγ and IL-4, respectively. We then evaluated M1 and M2 mRNA gene expression. *Epha4* transcript was not present in KO-derived monocyte/macrophages but expressed in WT MΦ, M1, and M2 cells. Interestingly, we found *Epha4* was significantly downregulated following M1 but not M2 polarization suggesting it may play a key role in M2 function (Fig. 5e). Next, we evaluated how the loss of *Epha4* influenced the M1 and M2 gene profile. M1-polarized KO cells showed a significant reduction in *CD86*, *Il12p-40*, *Ccr2*, and *Mcp1* (Fig. 5f–h, l) while M2-polarized cells showed a significant increase in *Arg1*, *Tie2*, and *Angpt2* compared to WT cells (Fig. 5i–l). In addition, we found *cx3cr1* was reduced in MΦ KO cells compared to WT, and *Il1ra* was reduced in KO M1 monocytes, while no differences were seen in *c-Myc*, *Erg2*, and *Tnfr1/Tnfr2* (Additional file 3: Figure S3). These findings further confirm a novel role for EphA4 in regulating the polarization state of monocyte/macrophages.

## Discussion

Eph receptor signaling plays a central role in the disease of CNS [5, 6, 49–52]. The current study reveal a novel role for this axon growth and guidance molecule in regulating the pro-inflammatory state of monocyte/macrophage and mediating tissue damage following TBI. We demonstrate EphA4 is upregulated in the cortex within hours of CCI injury and on CX3CR1-expressing infiltrated and/or resident monocyte/macrophages in the peri-lesion cortex. We also show that inhibition of the EphA4 receptor [20, 53] provides significant tissue protection in a murine model of cortical contusion injury which mimics the effects observed in global EphA4^−/−^ mice. Gene expression analysis of the cortex and peripheral immune cell response indicates that blocking EphA4 following peptide inhibition attenuates a pro-inflammatory milieu while promoting a pro-resolving state. These findings were mimicked in EphA4 bone marrow chimeric KO mice indicating loss of EphA4 is neuroprotective, in large part, by regulating the peripheral immune system. These results show a novel mechanism by which inflammation is regulated by Eph receptor signaling. In vitro, we also show that EphA4 inhibition blunts the LPS-induced response of cultured monocyte/macrophages and endothelial cells, shifting them towards a pro-resolving rather than pro-inflammatory phenotype, potentially via p-AKT signaling [54]. These novel findings demonstrate EphA4 negatively regulates acute TBI outcome by mediating the pro-inflammatory milieu.

Monocyte infiltration and inflammation is a major component of secondary brain injury and has been a target of treatment aimed at limiting TBI-induced disability [55, 56]. Trauma initiates both local CNS and systemic peripheral inflammation processes [57–59]. Previous studies have implicated EphA4 in chronic glial scar formation and are upregulated in acute closed-head injury in humans and non-human primates [12, 60]. We also find a significant acute increase in EphA4 expression in the ipsilateral cortex within hours following CCI injury, which correlates with the induction of inflammation in the brain following TBI [57]. The inflammatory response is a key driver in the pathogenesis of TBI [61]; however, the mechanisms underlying its influence remain poorly understood. The failure of anti-inflammatory drugs to improve outcome in a human clinical trial suggests a more complex role of inflammation which may be reflective of regional (resident vs peripheral), phenotypic (M1 vs M2), and time-dependent differences that occur in response to TBI. A better understanding of these changes is needed in order to restore immunologic balance.

Cytokines are induced in a time-dependent manner in the human brain and arterial plasma including TNF, IL-1, IL-6, IL-8, IL12p70, MCP-1, IL-10, and VEGF [57, 62, 63]. IL-6 and IL-8 are increased in serum following injury and correlate with unfavorable outcomes of human patients [64, 65]. It has also been previously shown that IL-6 blockage after TBI decreased motor coordination deficits in TBI/hypoxia models [66]. We demonstrated decreased levels of *Il6* mRNA in the blood and brain of VTM-EEKK- and KYL-treated mice, which has been correlated with better patient outcome in humans [67]. We also demonstrated that VTM and KYL peptide were capable of attenuating the IL-8 homolog *Cxcl1* mRNA levels with acute inflammation in cultured macrophages. It is also important to note that EphA4 inhibition or deficiency consistently decreased *Mcp1* signaling throughout our study, which is a key signaling molecule in monocyte infiltration into the brain through CCR2 [68]. The attenuation of pro-inflammatory gene expression following in vitro LPS stimulation after loss of EphA4 or peptide inhibition of EphA4 suggests that EphA4 may be responsible for increased inflammation and secondary damage following TBI. Our findings suggest that EphA4 inhibition may shift the inflammatory response towards a pro-resolving state as shown by the differential changes in the expression of *Il-6*, *Il-8*, and *Il-12* and *TNF* compared to *Il10*, *Arginase-1*, *Angpt2*, and *Tgfβ*. Some of the mRNA level discrepancies between KYL and VTM treatments may be due to KYL promiscuity, leading to slightly different responses between the two inhibitors. However, further time- and dose-dependent evaluation is needed as these differences may be related to the length of binding time and potential internalization of peptide/receptor complexes. Nonetheless, our in vitro studies expand our understanding of the acute pro-inflammatory role of EphA4 and suggest the negative effects of EphA4 activation following CCI injury may be the result of its phenotypic control over peripheral-derived monocytes. Surprisingly, KO BMCs isolated from the injured cortex did not show greater differences in pro-inflammatory genes such as *Il6* compared to WT cells. Given the gene expression was evaluated sub-acutely, it is possible we overlooked the early pro-inflammatory activation of infiltrating immune cells and instead overserved the enhanced pro-resolving state of the cells.

The clinical importance of pro-resolving gene expression is evident in human plasma levels of the angiopoietin/Tie2 axis, which has been shown to be a predictive biomarker for vascular integrity and outcome following TBI [69]. We also found that once EphA4-null monocyte/macrophages are polarized to M1 pro-inflammatory phenotype, they exhibit less *CD86*, *IL-12p40*, *Ccr2*, and *Mcp1*. CD86 is a key glycoprotein expressed in macrophages that activate naive T cells, contributing to inflammatory signaling in other cell types, suggesting beneficial cross talk may also be possible among monocyte/macrophages and infiltrating T cells in the TBI milieu. It is also important to note a key marker and inflammatory cytokine IL12p40 is decreased in polarized EphA4-KO cells implying less inflammatory behavior once polarized to pro-inflammatory. Reduced MCP1/CCR2 signaling observed in EphA4-KO M1 monocyte/macrophages in vitro and in vivo may also explain decreased inflammation and infiltration of GFP+ immune cells in EphA4 chimeric mice. Moreover, once polarized to an anti-inflammatory phenotype, cultured EphA4-KO monocyte/macrophages exhibit higher *Arg1*, a prominent anti-inflammatory mediator, and increased *Tie2* and *Angpt2* expression compared to WT cells. While vascular Tie2 and Angpt2 expression are both necessary for vascular stability, which would be beneficial in the TBI milieu [69, 70], Angpt2 has also been shown to skew the phenotype of Tie2-expressing monocytes (TEMs) towards M2-like state [37]. Interestingly, we found the KO BMCs isolated from the injured cortex, and KO M2-skewed cells cultured in vitro displayed increased Tie2 and Angpt2 expression suggesting the loss of peripheral immune-specific EphA4 may increase the presence/numbers of (TEMs) or increase the pro-resolving functional characteristics TEMs in the injured milieu. However, given there is evidence that Tie2 may regulate the pro-inflammatory activation of human macrophages [71, 72], additional studies are needed to further explore whether these effects are cell and/or context dependent. Additional findings also demonstrate Tie2 expression and function on human neutrophils regulate their chemotaxis and viability [73, 74]. Further elucidation of these pathways, including analysis of NFkB, is needed to expand our mechanistic understanding of EphA4’s role in the peripheral immune cell response to TBI. In addition, their role in regulating brain resident-derived immune cell responses, such as microglia activation, and the potential role of EphA4 on these neuroimmune cells are needed.

While the controversial role of peripheral immune activation in brain trauma remains under investigation, our novel findings demonstrate the well-known axon guidance molecule, EphA4, is upregulated within hours of TBI and plays a substantial role in the neuro-immune milieu. We have also identified EphA4 as a novel regulator of the monocyte/macrophage inflammatory response following LPS stimulation and TBI. This newly identified regulator of neuroinflammation expands our knowledge of the key players that may be involved in fine-tuning the inflammatory profile in the brain necessary for tissue homeostasis.

## Conclusion

Traumatic brain injury (TBI) induces a complex cascade of events that elicits neural tissue damage and functional deficits. The extent of inflammation, both peripheral and CNS-derived, plays a major role in the outcome from TBI. The current findings implicate a novel role for EphA4 receptor tyrosine kinase in mediating the pro-inflammatory, neurotoxic milieu in response to cortical trauma. It is revealed that peripheral immune-derived EphA4 provides overzealous inflammatory signals that are detrimental to neural tissue survival, which we suggest may be regulated in part by monocyte/macrophage polarization states. These findings expand our knowledge of the mechanism(s) underlying inflammation in TBI and provide a framework for future investigations into immune cell type-specific control of neural dysfunction in CNS disorders.

## Supplementary information

**Additional file 1.** **Figure S1.** Data analysis of PhosphoArray analysis of cultured monocyte/macrophages. A full summarized antibody list against relevant total proteins and protein phosphorylation sites tested. Data was analyzed by Full Moon Inc., using GenePix Pro software. Data is expressed as fold change of KO cells relative to WT untreated (UNT) or WT LPS. Any fold change above 1.5 and below 0.5 were considered significant with A 95% CI was used to quantify the precision of the phosphorylation ratio based on the analysis of the six individual replicates. (TIF 8552 kb)

**Additional file 2.** **Figure S2.** Effects of EphA4 peptide inhibition on brain-derived endothelial cell inflammatory response to LPS and p-AKT expression. (A) Relative mRNA expression of pro-inflammatory genes following 4 hrs LPS-stimulation of WT ECs in the presence of VTM-EEKK and KYL compared to vehicle control. (B) Representative Odyssey IR images of western blot analysis. (C-F) Quantified EphA4 protein expression, p-AKT/AKT, p-ERK/ERK, and p-Cx43/Cx43, normalized to β-actin, in treated WT ECs. *P < 0.05, **P < 0.01, ***P < 0.001, ****P < 0.0001 compared to vehicle control treated WT ECs. (TIF 28937 kb)

**Additional file 3.** **Figure S3.** Inflammatory gene expression analysis of cultured MΦ, M1 and M2 WT and KO monocyte/macrophages. Monocyte/macrophages were polarized to M1 or M2 using 5 ng/mL M-CSF and by IFNγ or IL-4 treatment, respectively. MΦ were treated with 5 ng/mL M-CSF only. (A-B) EphA4-KO monocyte/macrophages exhibit differential expression of *Cx3cr1* and *Il1ra* inflammatory receptors upon polarization. (C-F) EphA4-KO monocyte/macrophages showed no difference in M2 markers *Erg2*, and *c-myc* or inflammatory receptors, *Tnfr1* and *Tnfr2* once polarized. All data was normalized to WT MΦ levels. **P < 0.01, ***P < 0.001 compared to WT cells. (TIF 14385 kb)

## Data Availability

Datasets analyzed during the study are available from the corresponding author on reasonable request.

## Abbreviations

Angpt: Angiopoeitin
IL: Interleukin
LPS: Lipopolysaccharide
M1: M1-like macrophage
M2: M2-like macrophage
TBI: Traumatic brain injury
TNF: Tumor necrosis factor alpha
