# Supplementary figures and images for: Peripheral loss of EphA4 ameliorates TBI-induced neuroinflammation and tissue damage
Source: J Neuroinflammation. 2019 Nov 11;16:210. doi: 10.1186/s12974-019-1605-2 (PMC6844068; doi:10.1186/s12974-019-1605-2)

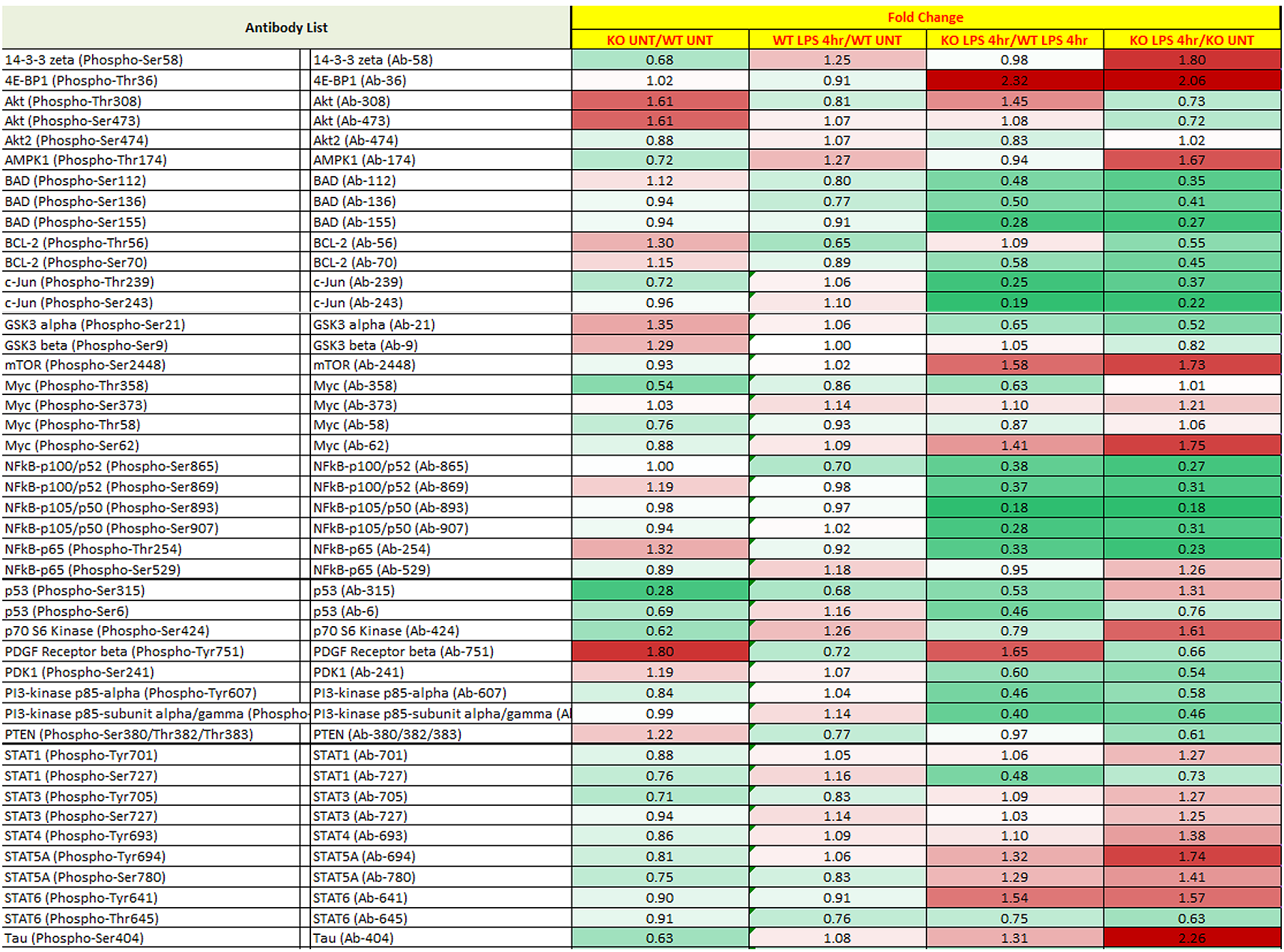

Supplement: Supplementary file 1 — Additional file 1. Figure S1. Data analysis of PhosphoArray analysis of cultured monocyte/macrophages. A full summarized antibody list against relevant total proteins and protein phosphorylation sites tested. Data was analyzed by Full Moon Inc., using GenePix Pro software. Data is expressed as fold change of KO cells relative to WT untreated (UNT) or WT LPS. Any fold change above 1.5 and below 0.5 were considered significant with A 95% CI was used to quantify the precision of the phosphorylation ratio based on the analysis of the six individual replicates. (TIF 8552 kb) [file 12974_2019_1605_MOESM1_ESM.tif]

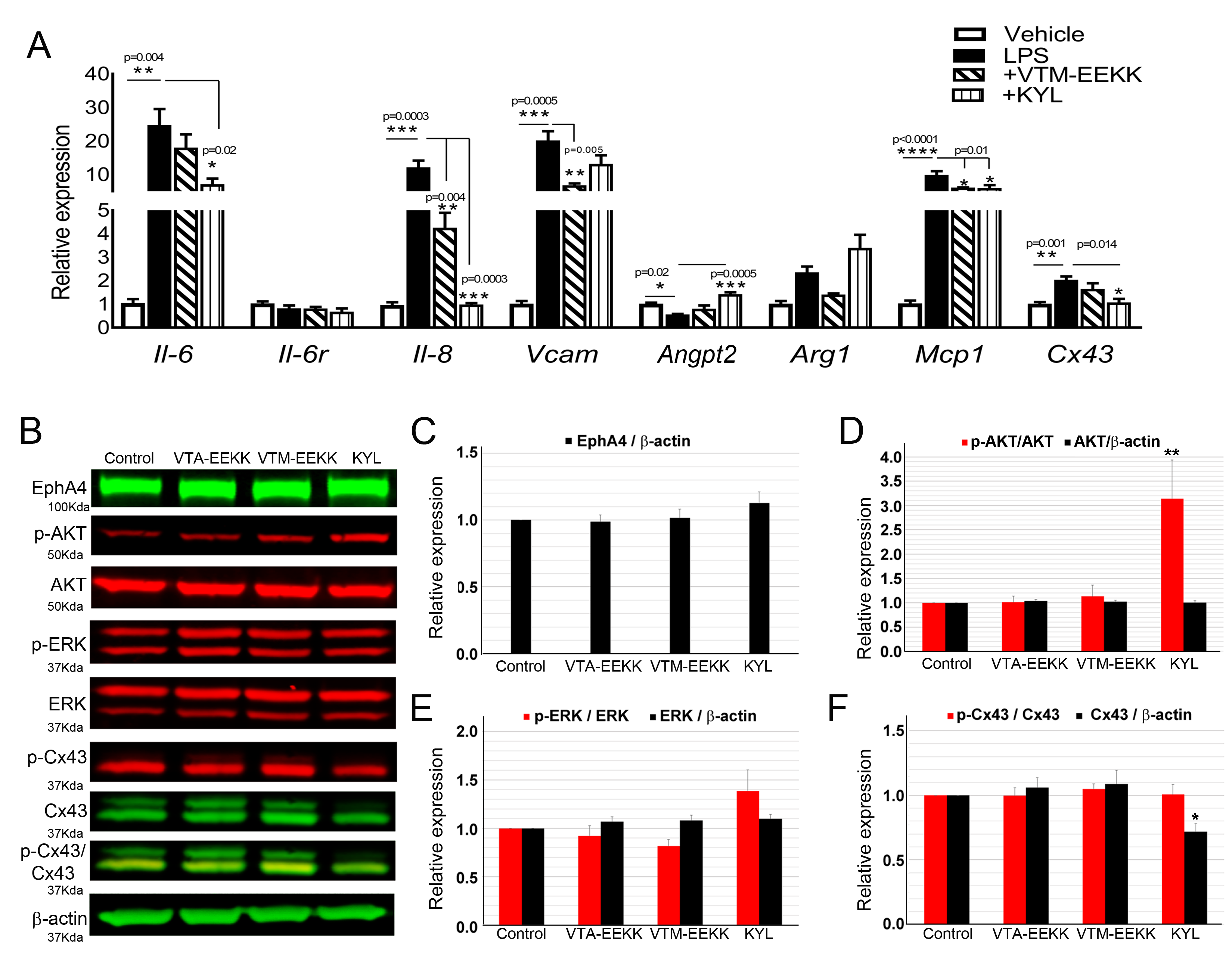

Supplement: Supplementary file 2 — Additional file 2. Figure S2. Effects of EphA4 peptide inhibition on brain-derived endothelial cell inflammatory response to LPS and p-AKT expression. (A) Relative mRNA expression of pro-inflammatory genes following 4 hrs LPS-stimulation of WT ECs in the presence of VTM-EEKK and KYL compared to vehicle control. (B) Representative Odyssey IR images of western blot analysis. (C-F) Quantified EphA4 protein expression, p-AKT/AKT, p-ERK/ERK, and p-Cx43/Cx43, normalized to β-actin, in treated WT ECs. *P < 0.05, **P < 0.01, ***P < 0.001, ****P < 0.0001 compared to vehicle control treated WT ECs. (TIF 28937 kb) [file 12974_2019_1605_MOESM2_ESM.tif]

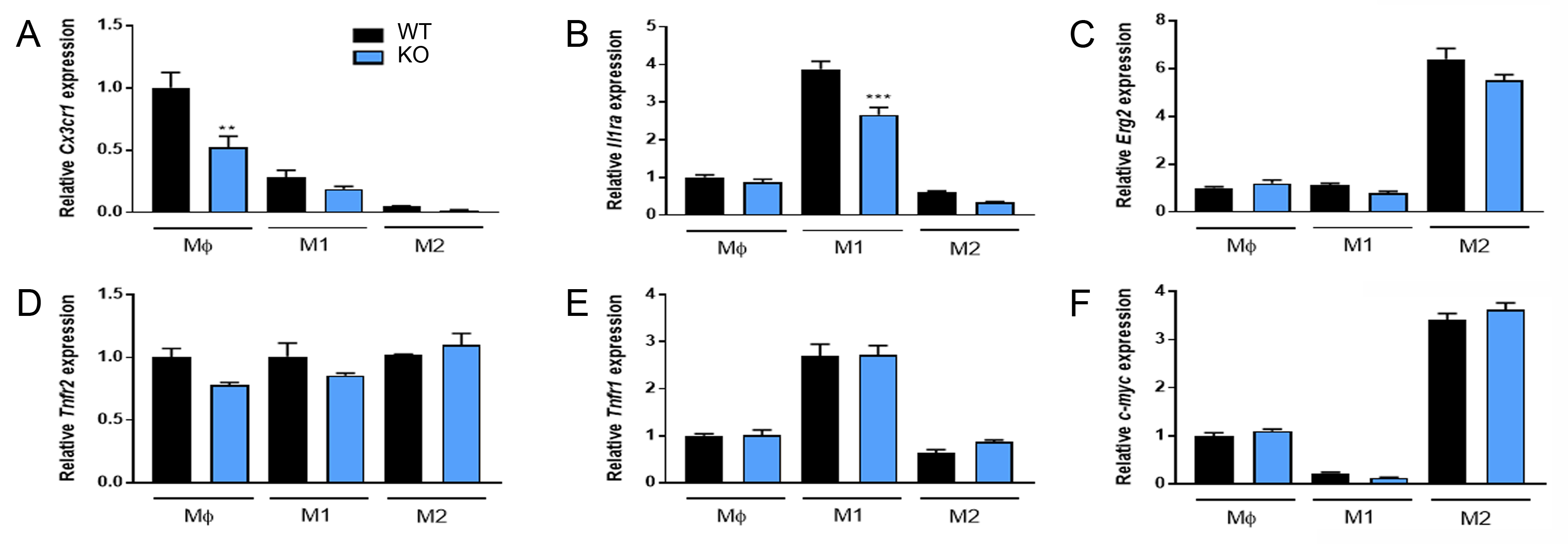

Supplement: Supplementary file 3 — Additional file 3. Figure S3. Inflammatory gene expression analysis of cultured MΦ, M1 and M2 WT and KO monocyte/macrophages. Monocyte/macrophages were polarized to M1 or M2 using 5 ng/mL M-CSF and by IFNγ or IL-4 treatment, respectively. MΦ were treated with 5 ng/mL M-CSF only. (A-B) EphA4-KO monocyte/macrophages exhibit differential expression of Cx3cr1 and Il1ra inflammatory receptors upon polarization. (C-F) EphA4-KO monocyte/macrophages showed no difference in M2 markers Erg2, and c-myc or inflammatory receptors, Tnfr1 and Tnfr2 once polarized. All data was normalized to WT MΦ levels. **P < 0.01, ***P < 0.001 compared to WT cells. (TIF 14385 kb) [file 12974_2019_1605_MOESM3_ESM.tif]
